# Sensitization to cereals and peanut evidenced by skin prick test and specific IgE in food-tolerant, grass pollen allergic patients

**DOI:** 10.1186/2045-7022-1-15

**Published:** 2011-12-09

**Authors:** Maria Martens, Heidi J Schnoor, Hans-Jørgen Malling, Lars K Poulsen

**Affiliations:** 1 Allergy Clinic, Copenhagen University Hospital, Gentofte, Denmark

**Keywords:** Cereals, cross-reaction, diagnosis, food challenge, grass pollen allergy

## Abstract

**Background:**

The botanical relation between grass and cereal grains may be relevant when diagnosing food allergy to cereals. The aim was to investigate the diagnostic specificity of skin prick test (SPT) and specific immunoglobulin E (sIgE) tests to cereals and peanut in grass pollen allergic subjects without history of, and clinically reactions to foods botanically related to grass.

**Methods:**

70 subjects (41 females; mean age 32 years) and 20 healthy controls (13 females; mean age 24 years) were tested by open food challenge (OFC) with cereals and peanut. SPT and sIgE both with Immulite^® ^(Siemens) and ImmunoCAP^® ^(Phadia) to grass and birch pollen, cereals, peanut and bromelain were performed.

**Results:**

Of the 65 OFC-negative subjects 29-46% (SPT, depending on cut-off), 20% (Immulite) and 38% (ImmunoCAP) had positive results to one or more of the foods tested. Controls were negative in all tests. Cross-reactive carbohydrate determinants (CCD) as evidenced by reaction to bromelain could explain only a minority of the measured IgE-sensitizations.

**Conclusion:**

Grass pollen allergic patients with documented food tolerance to cereals and peanut may express significant sensitization. False-positive cereal or peanut allergy diagnoses may be a quantitatively important problem both in routine clinical work and epidemiological studies.

## Introduction

Wheat IgE-mediated allergy manifests itself as food allergy [1] and as occupational inhalant allergy (Baker's asthma) [2] and identical allergens seem to be responsible in both allergies, although their relative importance differ [3,4]. It is a general clinical experience that also patients without these diseases may display positive skin test or immunoglobulin E (IgE) towards wheat and the cross-reactivity between grass pollen and cereals may have an impact on the specificity of the diagnostic tests [1,5-7].

In a non-published retrospective study performed in our department we found a large number of positive reactions to cereals in specific IgE (sIgE) tests in grass pollen allergic patients claiming to tolerate cereals, when interviewed by telephone. Also Jones *et al*. [8] found clinically non-relevant reactions to cereals in grass pollen allergic patients. Low specificity of cereal-related diagnostic tests is a particular problem since grass pollen allergy is very prevalent compared to true cereal allergy, but studies of grass pollen allergic subjects with clinical tolerance to cereals confirmed by food challenge are scanty. False positive tests may result in overestimation of cereal allergy prevalence in epidemiological studies, and on the individual level lead to unwarranted dietary restrictions or unnecessary double-blind, placebo-controlled food challenges (DBPCFC).

Our aim was to investigate the diagnostic specificity, i.e. number of false positives, of skin prick test (SPT) and sIgE tests to cereals and peanut in a group of grass pollen allergic subjects without history of and clinically reactions to foods botanically related to grass. This was done in a prospective study where tolerance to cereals and peanut in grass pollen allergic subjects was verified by a negative food challenge and the correlation between two different sIgE tests was investigated. While botanically unrelated to cereals, we also looked into the cross-reactivity between peanut and grass pollen. Studies have shown a clinically irrelevant association between grass and peanut [9-12], and we know that a positive test to peanut may alarm patients and clinicians.

## Materials and methods

### Study population

Recruitments were made by advertising in local newspapers for persons with a history of grass pollen allergy but no clinical reaction including exercise-induced anaphylaxis to cereals and peanut. Healthy control persons were also recruited. Inclusion criteria were: Age 18-60, history of allergy to grass pollen, positive SPT and sIgE (ImmunoCAP) level to grass pollen ≥ 0.70 kU_A_/L. No allergy related medicine was allowed for at least five days before the visits and all subjects must have a FEV_1 _≥ 80% of the expected value. Exclusion criteria were: pregnancy, breast-feeding, known allergies to cereals or peanut. Neither previous nor ongoing grass immunotherapy, nor allergy to other inhalant allergens or foods cross-reacting with birch constituted exclusion criteria.

The local Ethics Committee approved the study (H-D-2008-072) and informed consent was obtained from all subjects.

### Food challenge

Since the suspected outcome of challenges was confirmation of tolerance these were performed openly (OFC). Raw wheat, rye, barley, oat, maize, rice (all as flakes) and salted peanuts were administered in dose of 25 g each; all seven foods were consumed directly after one another, producing a total meal dose of 175 g and were consumed within half an hour. Addition of sugar, cow's milk, or soymilk was allowed. There were no restrictions regarding the diet in the days before the challenge. All subjects were observed for 2 hours, and any subjective and/or objective symptoms were recorded. Giving the test meal as single dose, with all seven foods without intervals, prevented us from further examining the positive reactions, and to identify to which food or threshold dose they occurred. For some the large dose might represent a large meal and could cause reactions they were not used to; e.g. satiety-related symptoms. Known asthmatics were monitored by FEV_1 _during the observation period.

### Skin prick test

Both allergics and controls underwent SPT with commercial extracts (Soluprick^®^, ALK-ABELLO, Hørsholm, Denmark) for a panel of common inhalation allergens (Table 1). Freshly made suspensions of flour and saline (1:10) were used for SPT with cereals (wheat, rye, oats, barley, maize and rice). SPT with peanut was made as prick-prick [13]. SPT was performed in duplicates on the volar aspect of the forearm including positive control (histamine dihydrocloride (10 mg/ml)) and negative control (Soluprick solution). The area of the weal was outlined with a pen, transferred to a record form and scanned [14]. For the standardized extracts of inhalation allergens the cut off was 7 mm^2^, corresponding to a wheal diameter of 3 mm, whereas for the foods no cut off was applied. There were no reactions to the negative control and none with dermographism. The results were expressed as the mean of the duplicates.

**Table 1:** Patients and controls demographics, sensitization status to inhalant allergens and asthma status.

	Allergic subjects	Controls
Mean age (years)	32	24
- range	18-57	21-30
Females	38	13
Males	27	7
**No. of patients sensitized (skin prick test) to inhalant allergens:**		
Grass (inclusion criterion)	65	0
Birch	26	0
- history of birch pollen allergy	24	0
- plus allergy to foods cross-reacting with birch	14	0
Mugwort	20	0
Dog	34	0
Cat	24	0
Horse	2	0
D. pteronyssinus	26	0
D. farinae	17	0
Alternaria	9	0
Cladosporium	5	0
**Asthma status:**		
Seasonal	5	0
Perennial	6	0
Mean onset age (years)	17	-
Outgrown asthma	5	0

### Specific IgE

All subjects and controls were tested for sIgE antibodies according to the producers' instructions on both the CAP-FEIA (ImmunoCAP^® ^250, Phadia, Uppsala, Sweden) and the Immulite (Immulite^® ^2500 3 g Allergy, Siemens Healthcare Diagnostics, Deerfield, IL, US). The allergens tested were grass (g6), birch (t3), wheat (f4), rye (f5), oat (f7), barley (f6), maize (f8), rice (f9), peanut (f13) and bromelain (k202). All sample measurements were performed in duplicates and results > 0.35 kU_A_/l (expressed as mean) were considered positive.

## Results

### Study population and open food challenge

Seventy subjects and 20 controls were tested by OFC and five subjects had a positive challenge test and were therefore excluded. The five reactors had symptoms of oral allergy syndrome, feeling of weight on the chest, feeling of swelling in throat, itch in the scalp and palms and rhinitis. All symptoms were mild and subjective only and cleared within the observation period after antihistamine (n = 1), bronchodilator (n = 1) or spontaneously (n = 3). The five reacting subjects were offered further investigation of their reactions by a new challenge, but only one accepted. Four of the five with positive OFC had a positive SPT to one or more of the foods tested.

All 20 controls had a negative OFC and negative SPT and IgE-tests to all inhalants and foods. Patients and controls demographics, sensitization status to inhalant allergens and asthma status are listed in Table 1. Most (83%) of the 65 patients had experienced grass-pollen-related symptoms for more than five years.

### Skin testing with foods

**Figure 1:**
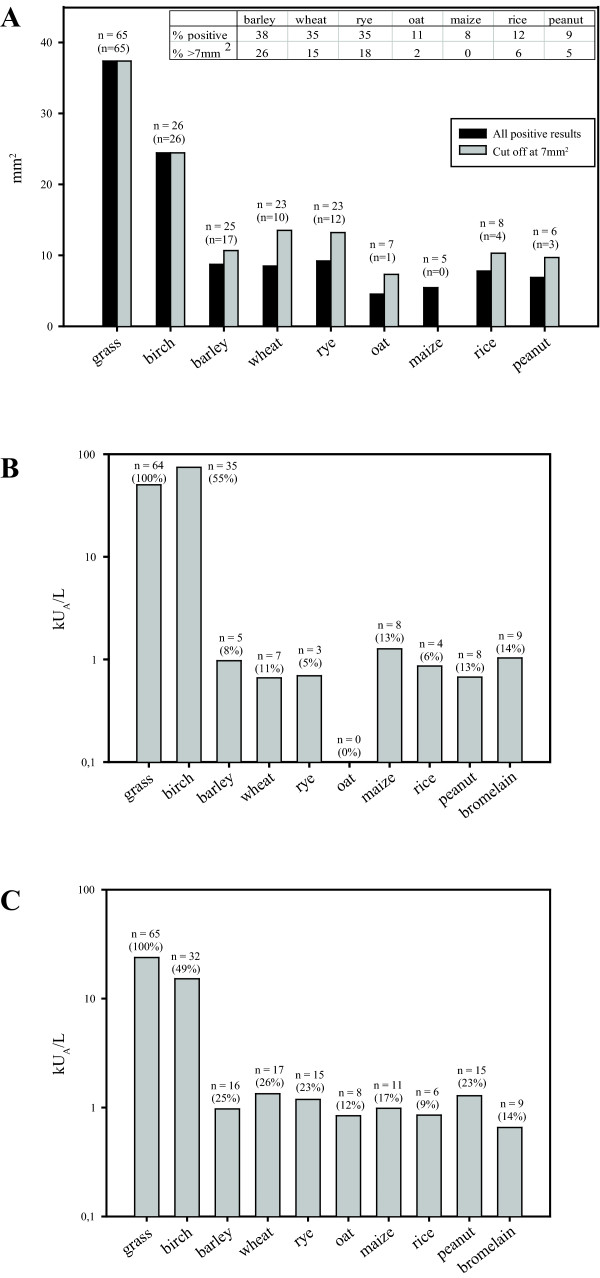
**Positive results of SPT and sIgE**. **A**. SPT mean wheal sizes of positive results. n = number of positives. Number of positive results > 7 mm^2 ^in parentheses. The inserted table shows percentages of positives with or without application of 7 mm^2 ^as cut off. **B**. Immulite, means of positive sIgE results > 0.35 kU_A_/L on log-scale. Percent positive in parentheses. **C**. ImmunoCAP, means of positive sIgE results > 0.35 kU_A_/L on log-scale. Percent positive in parentheses.

Thirty subjects (46%) had positive SPT to one or more of the foods tested. Excluding peanut, this gives 45% positives to any cereal. 9% reacted to peanut. The wheal size of SPT reactions to cereals and peanut was 5 times smaller than the wheal size for grass (Figure 1A). Subjects reacted most frequently to wheat, rye and barley (25 reactions to barley and 23 reactions to both wheat and rye, compared with 7, 5, 8 and 6 reactions to oat, maize, rice and peanut respectively). The number of positives would be remarkably reduced if the cut off at 7 mm^2 ^were applied, and the percentages being positive to at least one cereal would be reduced to 29%.

### Specific IgE by Immulite and ImmunoCAP

The number of subjects used for analyses of the Immulite results was reduced to 64 because of lack of serum from one subject who was therefore excluded from further analysis. The number of positive subjects was below 10 for all the foods and bromelain. In total 20% reacted to at least one of the foods tested. Twenty percent were positive to one or more cereals and 13% to peanut. In contrast to the SPT and ImmunoCAP results, less subjects sensitized to wheat, rye and barley were detected, nor did the Immulite identify any subjects sensitized to oat (Figure 1B).

In ImmunoCAP as in SPT, there was a majority reacting to wheat, rye and barley, but also many positive peanut reactions (Figure 1C). The percentages of positives were 38% to any of the foods tested, 32% to at least one cereal and 23% to peanut. The mean levels of sIgE to cereals and peanut were typically 40 times lower than to grass and birch both in Immulite and ImmunoCAP.

**Figure 2:**
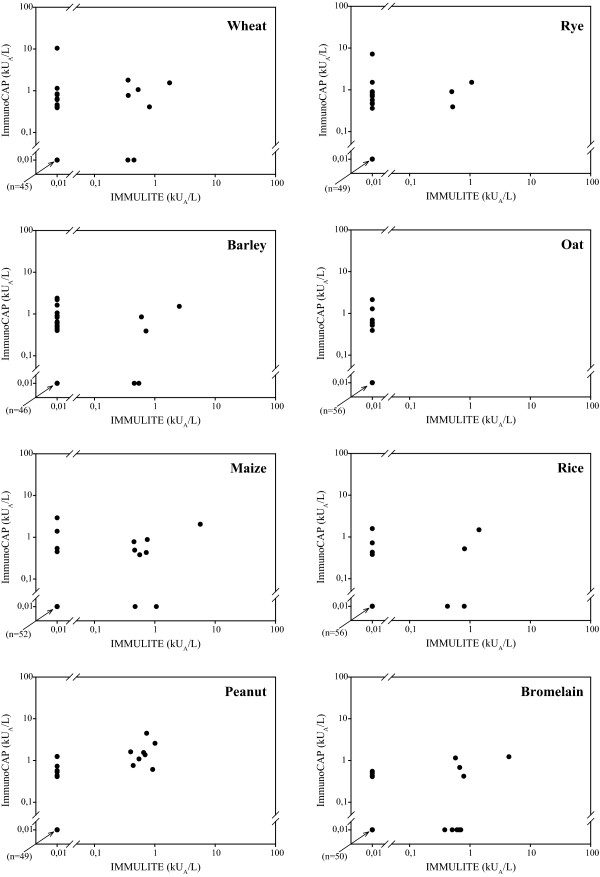
**Quantitative relation of sIgE against the different cereals, peanut and bromelain**. Immulite is plotted against ImmunoCAP for each cereal, peanut and bromelain on double-log-scale. Values depicted at 0.01 represent values < 0.35 kU_A_/L.

Immulite detected fewer positive reactions than ImmunoCAP. There was a low degree of correlation between the two tests, but those that correlated were in the same level around 1 kU_A_/L (Figure 2). As for rye, oat and peanut the Immulite did not identify any other than those identified by the ImmunoCAP. The concordance ranged from 0.77-0.91.

### Relationship between foods

**Figure 3:**
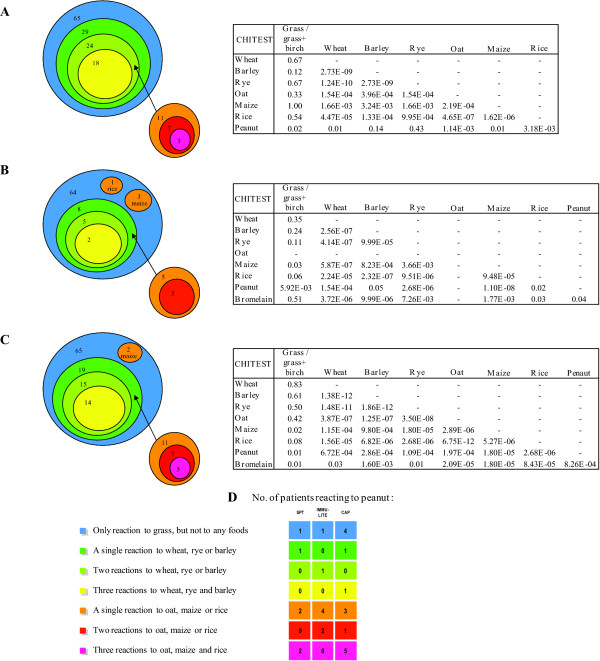
**Cluster diagrams and chi^2^-test results of relation between the cereals and peanut**. **A**. Skin prick test. **B**. Immulite. **C**. ImmunoCAP. **D**. Number of patients reacting to peanut in the different clusters.

Positive reactions to the different foods showed comparable patterns in SPT and sIgE results. These clinically non-relevant sensitizations could be divided into three groups. Group I: subjects sensitized to wheat, rye or barley; group II: subjects additionally sensitized to oat, maize or rice and group III: maize-sensitized individuals less dependent on other cereals (Figure 3A-C). In SPT (Figure 3A) 29 of the total of 65 subjects were positive to at least one of the three cereals; wheat, rye or barley. Twenty-four of these were positive to two or more of wheat, rye or barley and of these 18 subjects had a positive skin test to all three aforementioned cereals. Eleven subjects were further sensitized to one or more of the cereals in group II (oat, maize or rice), 7 to two or more and only 3 subjects were sensitized to all 6 cereals.

The Immulite (Figure 3B) and ImmunoCAP (Figure 3C) showed slightly different but similar patterns. To investigate whether the sensitization to the different foods was random, a chi-square test was performed (Figure 3A-C). All cereals were significantly correlated (p < 0.05). However, in SPT and ImmunoCAP there was a high association between wheat, rye and barley but in Immulite the cereals were moderately associated, some of this explained by the small number of positives. Co-sensitization to birch had an impact on sensitization to cereals, but no correlation was found except for maize in the two sIgE tests. Between peanut and birch there was a significant correlation. In Immulite 38% of those positive to both peanut and birch were also positive to bromelain, where it was 50% in ImmunoCAP. In both tests, those sensitized to all foods (and bromelain) were included in these percentages, indicating that it could be the result of sensitization to cross-reactive carbohydrate determinants (CCD) in these cases.

Reactions to peanut showed a less clear pattern in relation to the cereals. However, ImmunoCAP identified 15 subjects sensitized to peanut, but these appeared to be un-related to the clusters defined by the cereals (Figure 3D).

**Figure 4:**
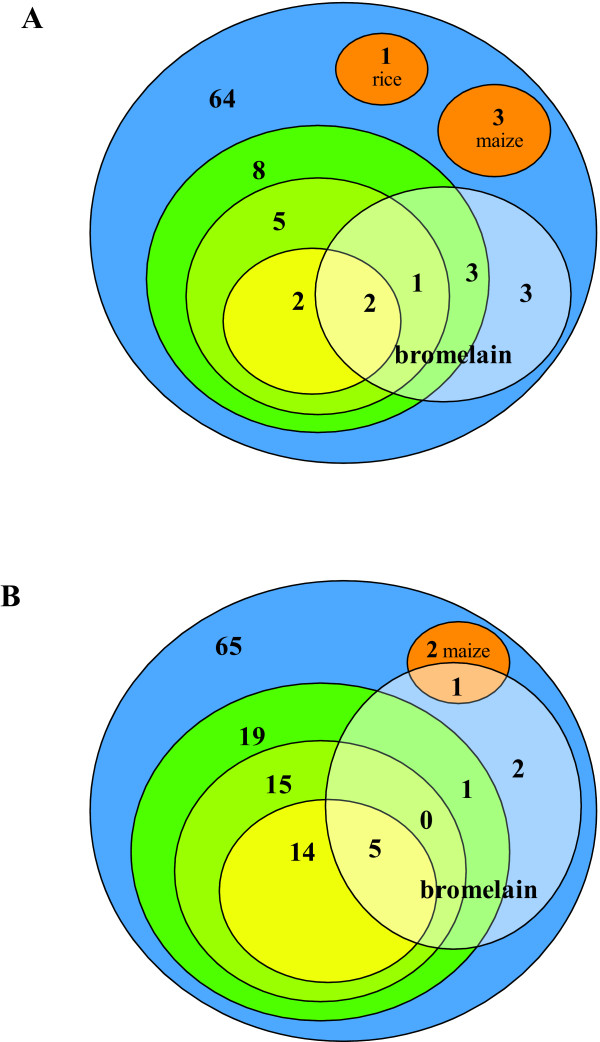
**Cluster diagrams showing the distribution of positives to bromelain**. Positives to bromelain are shown in the white shaded circles. Colour codes are as in Figure 3. **A**. Immulite. **B**. ImmunoCAP.

Bromelain was used as a marker for CCD that might explain some of the false-positive results in the grass pollen allergic subjects clinically tolerant to cereals and peanut. However, as shown in Figure 4A-B the subjects positive to bromelain were not only those sensitized to all the foods tested. The four subjects that were found positive to bromelain by both methods were the subjects that reacted to all seven foods in ImmunoCAP.

## Discussion

In this study we investigated sIgE-tests and SPT to cereals in grass pollen allergic subjects clinically tolerating cereals. This approach is rare possibly because there has been a strong focus on developing sensitive tests for food allergy, whereas the diagnostic specificity has attracted less attention. Cross-reactivity between pollen and foods is important for some allergens, but for others it is frequently not correlated to clinical food allergy [10,15-17].

Grass pollen allergy is one of the most frequent inhalant allergies with an estimated prevalence around 20% throughout the world, though it differs geographically [18,19]. With an estimated prevalence of grass pollen allergy of 20% of the general population our data could suggest that 9% would be positive in SPT for a cereal and 4-7% in the tests for sIgE. If such test results are used without knowledge of the primary grass sensitization and the low specificity of the diagnostic tests, serious consequences could occur. One aspect is a gross overestimation of cereal allergy prevalence in epidemiological studies, when sensitization is used as a proxy for clinical allergy and proper follow-up is not possible. In several epidemiological studies, strikingly many IgE positives to wheat were in fact found [20]. Another aspect relates to unwarranted dietary restrictions or needless DBPCFC in the daily practice. In the few studies investigating grass pollen allergy and cereal or peanut allergy, the food-allergy-status of the subjects has not been known with certainty [6,11,17,21] or the test groups were small [8,20].

In our study the positive reactions in both SPT and specific IgE tests all occurred in clinically tolerant subjects under the basic assumption that the result of OFC is a true representation of the clinical status. It might be criticized that we did not perform DBPCFC. Instead we used OFC, which we consider adequate since none of the subjects had any history of reacting to the foods tested, and the OFC is anyway recommended subsequent to a negative double-blinded challenge [22]. The reason for using raw cereals was that uncertainty still remains as to whether the allergenicity is reduced during heating, as seen for fruits and vegetables cross-reacting with birch pollen, even though Pastorello *et al*. [16] found that cooking did not induce significant alterations of the protein structure of the true allergens. It could have been interesting to have included a third group of wheat allergic subjects for comparison but this was outside our aim and not easily done due to the low prevalence [23]. Previous studies identified wheat allergens with molecular weight at 12-16 kDa [3,24] and the major grass pollen allergens in group 1 and 5 range from 27-38 kDa [25,26]. Despite this, common epitopes were identified in wheat and grass pollen as high molecular weight proteins [16,17]. This suggests that the wheat proteins responsible for wheat food allergy are not the same as the wheat proteins responsible for cross-reactions in grass pollen allergics [16].

In immunological terms the positive reactions are likely to be specific since none of the 20 healthy controls had any positive reactions in any of the tests. In the SPT we used fresh-made suspensions of flour and saline which could be a source of error. Due to the limited characterization of wheat allergens, commercially available extracts are difficult to compare [3,6], and freshly-made suspensions are used in the routine work at our Allergy Clinic. For the standardized inhalation allergen extracts used for SPT a cut off value of 7 mm^2 ^is applied, but is this valid for non-commercial extracts? We would argue that without better characterization and the unsatisfactory diagnostic accuracies of SPT and sIgE-test [1,6]; it is interesting to look at any reactions in the test, especially when all 20 healthy controls had negative SPTs-not a single reaction. The comparison of SPT and sIgE is hampered by the use of both non-commercial and commercial extracts. We used the cut off at 0.35 kU_A_/L, even though both Immulite and ImmunoCAP are able to measure IgE levels down to 0.1 kU_A_/L. Our data shows that a cut off lower than 0.35 kU_A_/L would not benefit the specificity. A cut off at 0.70 kU_A_/L (the inclusion criteria for grass pollen allergics) would improve the specificity, but the consequence for the sensitivity is unknown and was not studied in our design. The false positive rate-in clinical terms-was ranging from 0.20-0.46 of the three tests using the OFC results as the gold standard. The clinically irrelevant sensitization to cereals is often secondary to pollen sensitization showing in the level of positive reactions to grass pollen and wheat. We found that the mean SPT wheal size and the level of sIgE to cereals were much lower than to grass. Matricardi *et al*. [21] also found this significant difference in the level of sIgE. Furthermore they found that sensitization to wheat most frequently was secondary to pollen sensitization. They did not confirm the observed sensitization to foods by food challenge and therefore cannot conclude about the clinical relevance of these sensitizations. In 1995 Jones *et al*. [8] found that only 1 of 6 grass pollen allergic subjects tolerating cereals had SPT wheals to cereal grains > 3 mm, whereas all 6 had grass SPT wheals > 3 mm, supporting our assumption that the difference in level of sIgE or wheal size of SPT between the primary and secondary sensitization may indicate the clinical relevance. However, the difference in level of sIgE in general cannot predict the clinical relevance [27], as the cross-reaction to nuts in birch pollen allergic subjects is of high clinical relevance even though the level of sIgE to birch and nuts differ [28].

We saw a cluster of reactions to wheat, rye and barley more frequently than to oat, maize and rice. Wheat, rye and barley are taxonomically from the same tribe, where oat, maize and rice belong to three other tribes, with only oat belonging to the same subfamily as wheat, rye, barley and grass [8,26]. This may explain why we see this cluster, which was also observed by Jones *et al*. [8] with 41-57% positive to wheat, rye and barley compared with 23-28% positive to oat, maize and rice. We cannot compare these results directly because some of the patients in Jones' study suffered from wheat allergy.

We also included peanut in spite of the absence of close botanical relation with cereals or grass pollen. Mortz *et al*. [9] found that 96% of Danish school children that were sensitized to peanut also had a concomitant reaction to grass pollen. In accordance with our findings they also found a lower sIgE level to peanut than to grass pollen, in grass pollen sensitized subjects with no clinically relevant peanut hyper-sensitization. Guilloux *et al*. [11] investigated peanut allergic patients some of which were also grass pollen allergic, and found a median sIgE level to peanut > 100 kU_A_/L (Immulite) and 49 kU_A_/L (ImmunoCAP) in the peanut allergic patients, whereas the median peanut sIgE was 0.11 kU_A_/L and 0.68 kU_A_/L respectively, in grass hay fever patients tolerating peanuts. The cross-reaction could also be due to Bet v 1 homologues in peanut, since all the patients positive to peanut were birch pollen sensitized, except one in the ImmunoCAP.

Reactions to CCD offer a good explanation on the observed cross-reactivity between grass pollen and cereals and peanut because of limited clinical relevance of CCD in the majority of grass-pollen-allergic patients [12,29-31]. Our data with bromelain suggests that CCD might explain the reactions to cereals and peanut in only a few cases, however. It would be of interest to study this in detail by immunoblot assays and inhibition analyses. Constantin and co-workers performed a study of IgE-reactivitiy to components from grass pollen and wheat in different patient groups, of which was also included a grass pollen allergic, anamnestically food allergy-negative group of patients [32]. Interestingly, 65% of these patients reacted to wheat flour in ImmunoCAP, but only 24% of the patients reacted to a wheat component (profilin), leaving the majority of the false-positive reactions to wheat unexplained. One could speculate that procalcin-another grass pollen pan-allergen-could also participate as a non-clinical relevant allergen [33].

It could be anticipated that the progress in wheat allergy diagnostics where single allergens responsible for wheat food allergy and baker's asthma are being characterized [1,3,4,24,32-34] may improve the specificity of such tests compared with whole extracts.

In conclusion, grass pollen allergic patients with proven food tolerance may express significant sensitization (albeit depending on the test system) to cereals and peanut. Overall 45%, 19% and 32% reacted to one or more cereals in SPT, Immulite and ImmunoCAP, with wheat, rye and barley causing the most frequent reactions. Likewise, peanut caused 9%, 13% and 23% false positive reactions. Negative food challenges for exclusion of clinical allergy are occasionally required in routine clinical work and clearly in epidemiological studies.

## Abbreviations

SPT: skin prick test; OFC: open food challenge; FEV_1_: Forced expiratory volume 1^st ^second; sIgE: specific immunoglobulin E; CCD: cross-reactive carbohydrate determinants; DBPCFC: double-blind; placebo-controlled food challenge.

## Competing interests

The authors declare that they have no competing interests.

## Authors' contributions

MM carried out the clinical study, recruitment of study population, data acquisition and analysis, participated in sIgE measuring, designed the case report forms and drafted the manuscript. HJS was responsible for the preparation and conduction of the oral food challenges and participated in revising the manuscript. HJM had the clinical responsibility and participated in coordination of the clinical study and manuscript revising. LKP designed the study, wrote the protocol and participated in data analysis and manuscript revising. All authors have read and approved the final manuscript.
